# Ketogenic Diet for Preoperative Weight Reduction in Bariatric Surgery: A Narrative Review

**DOI:** 10.3390/nu14173610

**Published:** 2022-08-31

**Authors:** Luca Colangeli, Paolo Gentileschi, Paolo Sbraccia, Valeria Guglielmi

**Affiliations:** 1 Department of Systems Medicine, University of Rome Tor Vergata, 00133 Rome, Italy; 2 Obesity Medical Center, Policlinico Tor Vergata, 00133 Rome, Italy; 3 Department of Bariatric and Metabolic Surgery, San Carlo of Nancy Hospital, University of Rome Tor Vergata, 00133 Rome, Italy

**Keywords:** bariatric surgery, pre-operative weight loss, ketogenic diet, very-low-calorie ketogenic diet, VLCKD

## Abstract

Bariatric surgery (BS) is the most effective treatment in reducing weight and the burden of comorbidities in patients with severe obesity. Despite the overall low mortality rate, intra- and post-operative complications remains quite common. Weight loss before BS reduces surgical risk, but studies are inconclusive regarding which is the best approach to apply. In this review, we summarize the current evidence on the effect of a ketogenic diet (KD) before BS. All studies agree that KD leads to considerable weight loss and important improvements in terms of surgical risk, but populations, interventions and outcomes are very heterogeneous. KD appears to be a safe and effective approach to induce weight loss before BS. However, randomized controlled trials with better-defined dietary protocols and homogeneous outcomes are necessary in order to draw firm conclusions.

## 1. Introduction

Bariatric surgery (BS) is the most effective treatment in patients with severe obesity in terms of durable weight loss and the reduction of comorbidity burden and mortality [1,2].

Among the different surgical techniques, Roux-en-Y gastric bypass (RYGB) and sleeve gastrectomy (SG) are the most commonly used [3] and they are usually performed laparoscopically [4], but in these patients surgical laparoscopic procedures represent a challenge because of the thickness of the abdominal wall, the considerable accumulation of visceral adipose tissue and the increased liver volume [5]. As a matter of fact, despite the overall low mortality rate, surgical complications such as anastomotic leakage, bleeding and infections remain quite common (5–20%) [6]. The excess of visceral fat increases the risk of surgical complications and also increases the conversion rate and operative time [7]. For example, an enlarged liver and the accumulation of visceral fat may hamper the surgical field and this is the cause of the conversions in RYGB in approximately 50% of conversions cases [8]. Moreover, a large neck circumference (>44 cm) often results in difficult intubation and problems with mechanical ventilation [9].

It has been reported that immediate pre-operative weight loss can limit anesthesiological and surgical risks [10] and also seems to improve short- and long-term outcomes [11], but its role is still matter of discussion [12]. In 2016 the American Society for Metabolic and Bariatric Surgery stated that “insurance-mandated preoperative weight loss is not supported by medical evidence and has not been shown to be effective for preoperative weight loss before BS or to provide any benefit for bariatric outcomes” [13]. Indeed, there is a lack of multicenter, randomized, controlled trials on this specific topic [14], so most of the evidence on the effects of pre-operative weight loss come from retrospective studies. Thus, at present, guidelines do not provide any conclusive indication about pre-operative weight reduction [15,16].

Different approaches used to induce weight loss before surgery have been investigated, such as pharmacotherapy with glucagon-like peptide-1 receptor agonists (GLP-1 RAs) [17] or the combination of a hypocaloric diet with intragastric balloon placement [18], which significantly decreased the rate of conversions and complications in a case-control study [19].

However, diet is probably the most common approach in clinical practice and previous studies have already described the beneficial effect of a Mediterranean diet on weight loss before and after BS [20,21]. Some authors have reported that patients who show greater adherence to a diet before surgery are also those who lose more weight after surgery, for example, following a low-calorie diet (LCD) [22]. For patients at higher risk, in order to obtain more rapid weight reductions, a very-low-calorie diet (VLCD), characterized by a daily caloric intake of about 600–800 kcal, may be a valid option [23].

**Figure 1:**
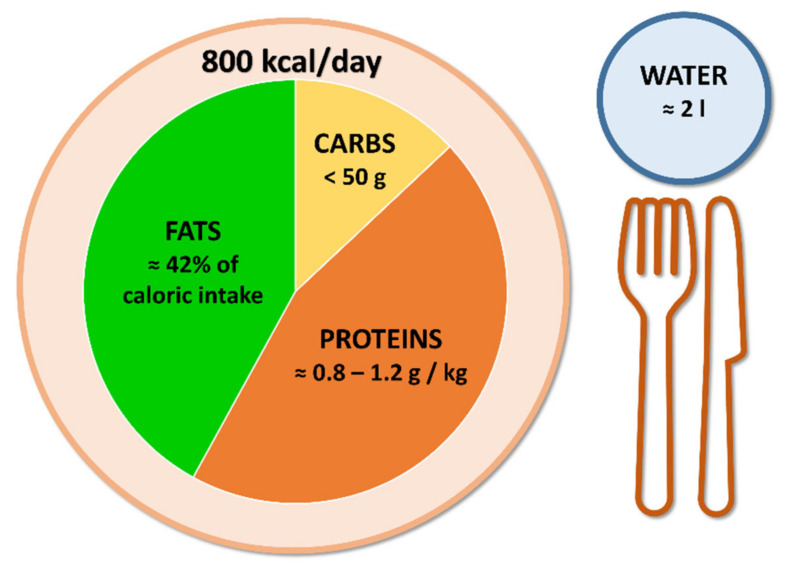
Very-low-calorie ketogenic diet principles.

Another interesting dietary approach that is more and more frequently used is a ketogenic diet (KD). KD is an “umbrella term” under which different low-carbohydrate dietary protocols are included. The common feature of these diets is that most of the caloric intake is derived from proteins and fats, inducing a fasting-like state with the development of physiological ketosis. For example, a very-low-calorie ketogenic diet (VLCKD) is characterized by a very important reduction in carbohydrate consumption (less than 50 g per day, providing approximately 13% of caloric intake), with adequate protein introduction (about 0.8–1.2 g for each kg of ideal body weight, providing approximately 45% of caloric intake) and a relatively increased consumption of fats (approximately 42% of caloric intake), with an average energy intake of 800 kcal/day [24] (Figure 1). KD is gaining growing popularity because patients usually report satisfaction with this nutritional approach, probably because ketone bodies have anorexigenic, euphoric and mood-stabilizing effects which lead to the reduction of hunger and a feeling of rapid satiety [25].

Originally used to treat epilepsy in children [26], KD has been demonstrated to be a valid tool for weight management in patients with obesity. Particularly, a recent meta-analysis [27] reported that VLCKD is associated with a mean BMI reduction of 3.25 kg/m^2^ (95% CI: 2.63 to 3.86) already at 1-month follow-up and up to 7.11 kg/m^2^ (95% CI: 5.38 to 8.84) after 12-month follow-up, which are also very encouraging results for individuals who are not planning to undergo bariatric surgery. Significant reductions have also been reported in glycated haemoglobin (HbA1c) of 0.43% (95% CI: 0.16 to 0.70) and in low density lipoprotein (LDL) cholesterol of 9.04 mg/dL (95% CI: 4.15 to 13.94), which makes VLCKD a valid nutritional strategy for cardiovascular disease prevention as well, although these results are not superior in comparison with other weight loss interventions.

The exact mechanisms by which KD induces its beneficial metabolic effects are still under discussion, but interest regarding its impact on gut microbiota is growing [28]. In a recently published study, 45 days of VLCKD led to a reduction in the relative abundance of Firmicutes and an increase in Bacteroidetes, particularly in VLCKD with whey protein [29]. The Firmicutes/Bacteroidetes ratio is usually increased in people with obesity and its reduction reflects a healthier intestinal homeostasis [30], although further studies are needed as other authors have reported contradictory results [31].

Overall, KD has proven to be a valid strategy to induce rapid weight loss [32] and its use before surgery, especially in short-time-available conditions, is particularly attractive [33].

The aim of this review was to summarize the current evidence on KD (with a particular focus on VLCKD) as a tool for pre-operative weight loss.

## 2. Concerns Regarding Pre-Operative Oxidative Stress

The risk that excessively rapid weight loss could cause a catabolic state and increase oxidative stress, with a negative impact on surgical outcomes [34], has been a major obstacle to studying the role of KD before BS. Conversely, preoperative administration of carbohydrates was demonstrated to reduce perioperative stress [35]. KD causes only a modest increase in ketone bodies, which reach levels far below those of diabetic ketoacidosis. Moreover, it has been demonstrated that KD can attenuate oxidative stress, probably thanks to the increased expression of mitochondrial uncoupling proteins and the consequent reduction in reactive oxygen species (ROS) production [36].

Leonetti et al. [37] were the first authors to address the role of a VLCKD in preparation for BS. To satisfy these safety issues, they proposed a sequential diet which they called “the OPOD (obese preoperative diet) regimen”, consisting of 10 days of VLCKD (daily energy intake of about 600 kcal, 15 g of carbohydrates, 80 g of proteins and 23 g of lipids), followed by 10 days of VLCD (daily energy intake of about 800 kcal, 55 g carbohydrates, same proteins and 30 g of lipids) and finally LCD (daily energy intake of 1100 kcal, with an increase in carbohydrates up to 145 g, 60 g proteins and 33 g lipids) until surgery. Fifty patients (31 females and 19 males, mean age 47.7 ± 11.2 years, mean BMI 53.5 ± 8.4 kg/m^2^) were enrolled in the study and compared to 30 patients (18 females and 12 males, mean age 43.3 ± 8.7 years, mean BMI 54.8 ± 9.4 kg/m^2^) who followed a standard LCD (1200 kcal/day) for the whole observation period. Body weight, waist circumference and neck circumference decreased significantly in the OPOD group (respectively, from 150.4 ± 26.3 kg to 137.6 ± 22.5 kg, *p* < 0.001; from 53.5 ± 8.4 to 49.2 ± 8.7 kg/m^2^, *p* < 0.001; and from 44.0 ± 3.3 cm to 41.1 ± 5.2 cm, *p* < 0.03), whereas there was no significant change in the control group. The OPOD group also registered an improvement in fasting plasma glucose levels, even in patients with type 2 diabetes mellitus taking antidiabetic medications. Regarding its safety in relation to liver and kidney functions, there were no significant modifications in the levels of creatinine, urea, uric acid, glutamic oxaloacetic transaminase, glutamic pyruvic transaminase, γ-glutamyl transferase or alkaline phosphatase. Ultrasound evaluation was performed and a mean 30% reduction in liver volume was found.

Altogether, the results of this trial were promising since they achieved significant weight loss without safety concerns, encouraging the study of KD before BS.

## 3. Micronutrient Deficiency

Another concern for candidates for BS is micronutrient deficiency (MD) [38]. Folate, vitamin D, thiamine, cobalamin, vitamin A, vitamin E, zinc, iron and selenium deficiencies are common in these subjects [39,40,41]. Micronutrient status should be checked before BS not only to optimize clinical conditions present at the time of surgery, but also because BS procedures may exacerbate pre-existing MD [42,43].

Pilone et al. [44] proposed a sequential diet regimen consisting of 10 days of a VLCKD (which they called a V-diet), followed by a hypocaloric scheme (called V-hypo) for the next 20 days, with a progressive increase in caloric intake. Multimineral and multivitamin supplements were added to the diet according to daily requirements [21]. One-hundred nineteen patients (75 females, 44 males, mean age 43.6 ± 9.8 years, mean BMI 41.5 ± 7.6 kg/m^2^) were included in the study. At the end of the treatment, weight, BMI and waist circumference were significantly decreased (weight: from 117.4 ± 27.3 kg to 101.6 ± 26.6 kg, *p* < 0.0001; BMI: from 41.5 ± 7.6 kg/m^2^ to 34.1 ± 5.2 kg/m^2^, *p* < 0.0001; waist: from 116.3 ± 5.0 cm to 107.0 ± 3.9 cm, *p* < 0.005). Body composition analysis showed a reduction in the fat mass percentage (−15.1%, *p* < 0.05) after the V-diet, which was also partially maintained after the V-hypo, whereas there was no significant reduction in the fat-free mass percentage (−6.5%, *p* = 0.076). These findings suggest that muscle preservation is one of the main advantages of KD. Ultrasound evaluation also showed an almost one-third reduction in liver volume and an improvement in steatosis patterns.

Similarly, Schiavo and colleagues [45] studied the effect of a 4-week preoperative ketogenic micronutrient-enriched diet in patients scheduled for BS. Micronutrient status (including vitamins A, D, E, C, thiamine, cobalamin, iron, zinc, magnesium, selenium and folic acid) was evaluated in a cohort of 27 subjects (10 males and 17 females). In the 4 weeks prior to surgery, the patients followed a ketogenic food plan (about 1200 kcal/day, consisting of 4% carbohydrates, 71% fats and 25% proteins) enriched with a supplement composition (Ketocompleat, MVMedical Solutions, Serravalle, Repubblica San Marino). All subjects obtained a significant reduction in body weight (males 10.3%, *p* < 0.001 and females 8.2%, *p* < 0.001) and in left hepatic lobe volume (−19.8%; 503 ± 61 cm^3^ vs. 627 ± 85 cm^3^, *p* < 0.001). Regarding micronutrients status, there was a clear improvement in patients with preoperative vitamin B12, folic acid, iron and zinc deficiencies.

Both these studies are limited by the absence of a control group; however, they have the merit of having highlighted the importance of evaluating micronutrient status before BS and optimizing the dietary intervention with multimineral and multivitamin supplements.

## 4. Pre-Operative Care of Obstructive Sleep Apnea Syndrome

Obstructive sleep apnea syndrome (OSAS) is one of the most common comorbidities in patients with severe obesity waiting for BS [46]. Continuous positive airway pressure (C-PAP) for a minimum of 4 weeks before surgery is recommended in these patients to reduce anesthesiologic risk [47].

Schiavo et al. [48] conducted a randomized trial of 4 weeks involving patients with severe OSAS scheduled for BS who were divided in two groups. The intervention group included 34 patients who received both C-PAP and a low calorie ketogenic diet (a food plan providing 1200 kcal/day, consisting of 4% carbohydrates, 71% fats and 25% proteins), whereas patients included in the control group (*n* = 36) received only C-PAP. The primary endpoint was an improvement in apnea-hypopnea index (AHI) scores [49]. AHI scores improved significantly in both groups, with no particular advantage in the intervention group compared with the control group (*p* = 0.863), but combining two preoperative strategies led to a significant improvement in body weight (from 143.6 ± 23.6 kg to 129.7 ± 23.7 kg, *p* = 0.0052), BMI (from 50.1 ± 5.9 kg/m^2^ to 45.3 ± 6.5 kg/m^2^, *p* < 0.001) and C-reactive protein (CRP) levels (from 6.12 ± 5.59 mg/L to 2.66 ± 2.57, *p* = 0.0161), in addition to a reduction in systolic and diastolic blood pressure, HOMA index and cholesterol levels.

A limitation of this study was that control group did not receive any nutritional indication, so these findings can be applied to diet-induced weight reduction in general and not necessarily to LCKD.

## 5. Evaluation of Surgical Outcomes

Up this point, it has been difficult to understand which results are generally provided by weight loss before BS and which are the additive benefits of a specific KD.

Albanese et al. [50] tried to answer this question by comparing weight loss and surgical outcomes in two groups of patients who followed two different kinds of diets in the 3 weeks preceding surgery: a very-low-calorie ketogenic diet (VLCKD) and a very-low-calorie diet (VLCD). One-hundred and seventy-eight patients were enrolled in this study (139 women and 39 men, mean age: 43 years). Patients’ preferences influenced the type of diet, so 72 patients followed VLCKD, whereas 106 patients preferred VLCD (consisting of three main meals and two snacks for a daily caloric intake of 800 kcal/day, provided by 0.8–1.5 g/kg/day of proteins, 80 g/day of carbohydrates and 15 g/day of lipids). Patients were informed that weight loss before surgery was mandatory and their adherence to both kinds of diet was high. After 3 weeks, absolute weight loss was better in the VLCKD group than in the VLCD group (5.8 ± 2.4 kg vs. 4.8 ± 2.5 kg, *p* = 0.008) but there was no significant difference in the percentage of excess BMI loss (%EBMIL, respectively, 10.4 ± 4.0 and 10.0% ± 5.6%, *p* = 0.658). All patients underwent laparoscopic sleeve gastrectomy. Mean operative times and hospital stays were comparable in the two groups, but drainage output was lower (141.2 ± 72.8 mL vs. 190.7 ± 183.6 mL, *p* = 0.032), post-operative hemoglobin levels greater (13.1 ± 1.2 mg/dL vs. 12.7 ± 1.5 mg/dL, *p* = 0.04) and the percentage of patients requiring a prolongation of hospital stay (more than the predicted 3 days) were lower (2.8% vs. 10.4%, *p* = 0.048) in the VLCKD group compered to controls.

The authors concluded that, despite the fact that VLCKD and VLCD produced a comparable %EBMIL, VLCKD had better surgical outcomes and they supposed that these advantages were not strictly related to surgical maneuvers (since the operative time was comparable between the two groups) but rather to a better metabolic and nutritional status that positively influenced tissue healing.

## 6. Current Evidence and Future Perspectives

The results obtained from the studies discussed here on KD before BS are summarized in Table 1.

Abbreviations: BMI, body mass index; CPAP, continuous positive airway pressure; CRP, c-reactive protein; F, females; HOMA, homeostasis model assessment; LCD, low-calorie diet; LCKD, low-calorie ketogenic diet; M, males; OPOD, obese pre-operative diet; VLCD, very-low-calorie diet; VLCKD, very-low-calorie ketogenic diet.

**Figure 2:**
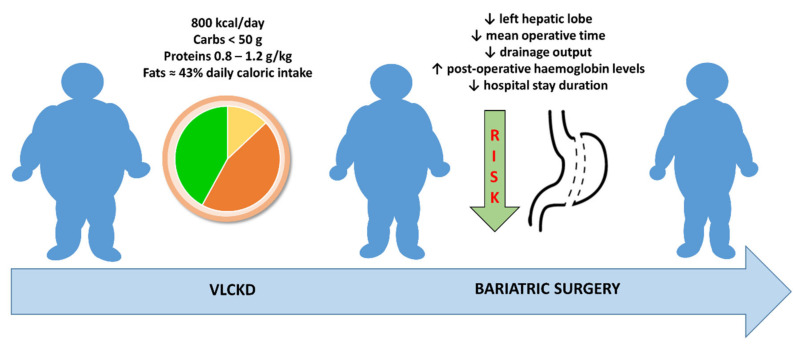
A very-low-calorie ketogenic diet can be a valid tool to induce weight loss before bariatric surgery, leading to the reduction of surgical risks.

Altogether, these studies confirm the usefulness of losing weight before BS and show the potential advantages of VLCKD (Figure 2), particularly if associated with micronutrient integration.

**Table 1:** Main findings of studies on KD before BS.

Reference	Population	Intervention Description and Duration	Control Group	Main Findings
Leonetti F et al., 2014 [37]	50 patients (31 F/19 M)	OPOD regimen: VLCKD for 10 days, VLCD for 10 days, LCD for 10 days	30 patients (18 F/12 M) standard LCD for 30 days	Reduction in BMI from 53.5 ± 8.4 kg/m^2^ to 49.2 ± 8.7 kg/m^2^ (*p* < 0.001); improvement in fasting plasma glucose levels; mean 30% reduction in liver volume; improvement of steatosis pattern.
Pilone V et al., 2018 [44]	119 patients (75 F/44 M)	Sequential diet regimen: VLCKD for 10 days, hypocaloric scheme for 20 days. In addition: multimineral and multivitamin supplements.	Absent	Reduction in BMI from 41.5 ± 7.6 kg/m^2^ to 34.1 ± 5.2 kg/m^2^ (*p* < 0.001); reduction of fat mass; preservation of fat-free mass; mean 30% reduction in liver volume; improvement of steatosis pattern.
Schiavo L et al., 2018 [45]	27 patients (17 F/10 M)	Ketogenic micronutrient-enriched diet for 4 weeks	Absent	Reduction in BMI from 46.9 ± 11.7 kg/m^2^ to 43.0 ± 13.4 kg/m^2^ (*p* < 0.001) in females; reduction in BMI from 44.5 ± 10.5 kg/m^2^ to 40.6 ± 6.5 kg/m^2^ (*p* < 0.001) in males; mean 19.8% reduction of left hepatic lobe; improvement in micronutrient status.
Albanese A et al., 2019 [50]	72 patients (60 F/12 M)	VLCKD for 3 weeks	106 patients (79 F/27 M) VLCD for 3 weeks	Total weight loss better in VLCKD than in VLCD group (5.8 ± 2.4 vs. 4.8 ± 2.5 kg, *p* = 0.008). Surgical outcomes: mean operative time slightly shorter in VLCKD group; percentage of patients requiring a longer-than-anticipated hospital stay lower in VLCKD group; lower drainage output and higher post-operative hemoglobin levels in VLCKD group.
Schiavo L et al., 2022 [48]	34 patients (12 F/22 M)	CPAP + LCKD for 4 weeks	36 patients (14 F/22 M) CPAP for 4 weeks	Apnea-hypopnea score improved in both groups; reduction in BMI (from 50.1 ± 5.9 kg/m^2^ to 45.3 ± 6.5 kg/m^2^, *p* < 0.001) was observed only in CPAP + LCKD group; reduction in CRP levels, blood pressure, HOMA index and cholesterol levels were observed only in CPAP + LCKD group.

F: females. M: males.

However, the current evidence has some limitations. First of all, the studies presented here are highly heterogeneous in terms of the intervention proposed: because of the small number of studies regarding this subject, we decided to take in consideration not only better-defined protocols such as VLCKD [51] but also more generic KDs. Similarly, studies evaluated different outcomes in addition to weight loss, varying from micronutrient status to drainage output. The designs of the studies and the absence of a control group did not allow us to obtain an understanding of the advantages exclusive to KD, other than those generically provided by weight loss. Particularly, regarding improved surgical outcomes, there could be many other explanations other than diet that were not properly analyzed. The number of patients included in these studies is limited and most of the patients were female. Finally, the absence of a follow-up excludes the possibility of investigating potential post-surgery effects. In fact, the reported outcomes should be considered associations rather than being causally related to KD. These issues can be addressed in future research, with the planning of randomized controlled trials with clearly defined dietary protocols, larger populations (with both genders equally represented), clinically relevant outcomes and longer follow-up times to evaluate potential effects that have not yet been investigated.

## 7. Conclusions

Preoperative weight reduction in patients scheduled for BS is a goal to be pursued as it may lead to many advantages, including reducing liver volume and visceral fat, lowering intra- and post-operative complications, shorter surgery times and reduced hospital stays.

It is also a way to motivate patients towards a lifestyle change, which BS does not exempt them from. It has been demonstrated that a ketogenic diet is a safe and effective way to induce weight loss, so it can be considered also in this preoperative context. However, there is a need for larger randomized controlled trials, with better-defined dietary protocols (e.g., VLCKD) and homogeneous outcomes, in order to draw firm conclusions. In addition, a longer follow-up could be useful in order to evaluate the long-term effects of preoperative weight loss.

## Data Availability

Not applicable.
